# Predictors of foot care behaviours in patients with diabetes in Turkey

**DOI:** 10.7717/peerj.6416

**Published:** 2019-02-08

**Authors:** Yasemin Yıldırım Usta, Yurdanur Dikmen, Songül Yorgun, İkbal Berdo

**Affiliations:** 1 Bolu Health School, Abant Izzet Baysal University, Bolu, Turkey; 2 Faculty of Health Sciences, Sakarya University, Sakarya, Turkey; 3 Izzet Baysal State Hospital, Bolu, Turkey; 4 Izzet Baysal Education and Research Hospital, Bolu, Turkey

**Keywords:** Behaviour, Diabetic foot, Diabetes mellitus, Foot-care

## Abstract

**Background:**

The management of diabetic foot complications is challenging, time-consuming and costly. Such complications frequently recur, and the feet of individuals with diabetes can be easily infected. The variables that predict foot care behaviours must be identified to improve foot care attitudes and behaviours. Thus, this study aimed to evaluate the predictors of foot care behaviours in individuals with diabetes and the role of these variables.

**Methods:**

This descriptive and analytic study was carried out between July 2015 and July 2016, and 368 outpatients with diabetes from a public hospital in Turkey were included. The participants had no communication, psychiatric or neurological problems and had been diagnosed with diabetes for at least 1 year. Foot care behaviour was the dependent variable and was evaluated with the foot care behaviour questionnaire. The relationship among foot care behaviours and sociodemographic characteristics, diabetes-related attitudes, disease perception, health beliefs and perceived social support was evaluated. Factors that independently predicted effective foot care behaviours were estimated via a linear regression analysis.

**Results:**

The foot care behaviour score of the participants was above average (54.8 ± 5.0). Gender (*t* = −2.38, *p* = 0.018), history of a foot wound (*t* = −2.74, *p* = 0.006), nephropathy (*t* = 3.13, *p* = 0.002), duration subscale of the illness perception scores (*t* = 2.26, *p* = 0.024) and personal control subscale of the health belief scores (*t* = −2.07, *p* = 0.038) were significant predictors of foot care behaviours. These variables, which provided model compatibility, accounted for approximately 22.0% of the total variance of the foot care behaviour score (*R* = 0.47, *R*^2^ = 0.22, *F* = 5.48, *p* ≤ 0.001).

**Discussion:**

Our results show factors that may affect diabetic foot care behaviours. Several of these factors prevent individuals from practising these behaviours. Further studies on the roles of barriers as predictors of foot care behaviours must be conducted.

## Introduction

Diabetes mellitus (DM) seriously and negatively affects the physical, mental and psychosocial well-being of patients (Aypak et al., 2012). The development of diabetic foot affects approximately 15.0–25.0% of patients at some point during their lives. Diabetic foot develops secondary to peripheral neuropathy and can lead to problems such as foot deformities, dry skin and skin cracking. These problems are the most common causes of diabetes-related hospital admissions, of which the most important is neuropathy (Sevinç, 2015; Aypak et al., 2012; Kurniawan & Petpichetchian, 2011).

The management of diabetic foot complications is challenging, time-consuming and costly. Such complications frequently recur, and the feet of individuals with diabetes can be easily infected. Every 30 s, someone in the world requires an amputation. Coping with these negative effects requires training and self-care skills. For this reason, individuals must develop self-care behaviours. In fact, healthy feet are easier to care for in comparison with feet damaged by the effects of diabetes (Sevinç, 2015; Aypak et al., 2012; Kurniawan & Petpichetchian, 2011).

The acquisition and application of this knowledge are equally important. In previous studies of diabetes, researchers have found a discrepancy related to the practise. Moreover, patients did not practice foot care behaviours despite their understanding of foot care behaviours (Neta, Da Silva & Da Silva, 2015; Aypak et al., 2012). In some studies, factors such as age, gender, level of education, duration of diabetes, education regarding diabetes-related complications, beliefs and perceptions of the importance of foot care, communication between patients and health personnel and lack of information about diabetic foot care significantly affected foot care behaviours (Gündüz & Karabulutlu, 2016; Aris, Blake & Adams, 2015; Seid & Tsige, 2015; Kartal & Özsoy, 2014; Li et al., 2014; Aypak et al., 2012; Broadbent, Donkın & Stroh, 2011). In addition, social support systems are beneficial for patients and are effective predictors of self-care, health promotion and diabetes management (Karakurt, Aşılar & Yıldırım, 2013; Park, Nguyen & Park, 2012). The performance of foot care behaviours, such as regular inspections of the sole and between the toes and the use of moisturising lotions, can prevent complications related to diabetic foot (Biçer & Enç, 2014; Chin et al., 2014; Li et al., 2014).

Developing and maintaining foot care behaviours is the most basic way of coping with diabetic foot. The predictor variables for foot care behaviours must be identified. In this way, improvements in modifiable variables can be achieved. However, some variables related to foot care behaviours have not been examined in previous studies in Turkey. Current studies often focus on demographic characteristics, lifestyle choices and knowledge and attitudes. No study has shown a relationship between foot care behaviours and health beliefs, social support and disease perception. In addition, diabetic foot care is a self-care requirement for individuals with diabetes (Ausili et al., 2018). Therefore, the variables examined in this study were determined according to previous studies on self-care behaviours (Ausili et al., 2018; Gurmu, Gela & Aga, 2018; Nie et al., 2018). This study examined demographic characteristics, lifestyle choices, knowledge, attitude, beliefs, health perception and social support variables. Considering the existing information, this study aimed to examine the role of the variables that predict foot care behaviours in individuals with diabetes.

## Materials and Methods

### Study design

This descriptive and analytic study examined the role of variables that predict foot care behaviours in individuals with diabetes.

### Sample and settings

The study included outpatients with diabetes in a public hospital between July 2015 and July 2016. Patients aged over 18 years with a diagnosis of diabetes but without communication, psychiatric or neurological problems were included in the study.

**Figure 1:**
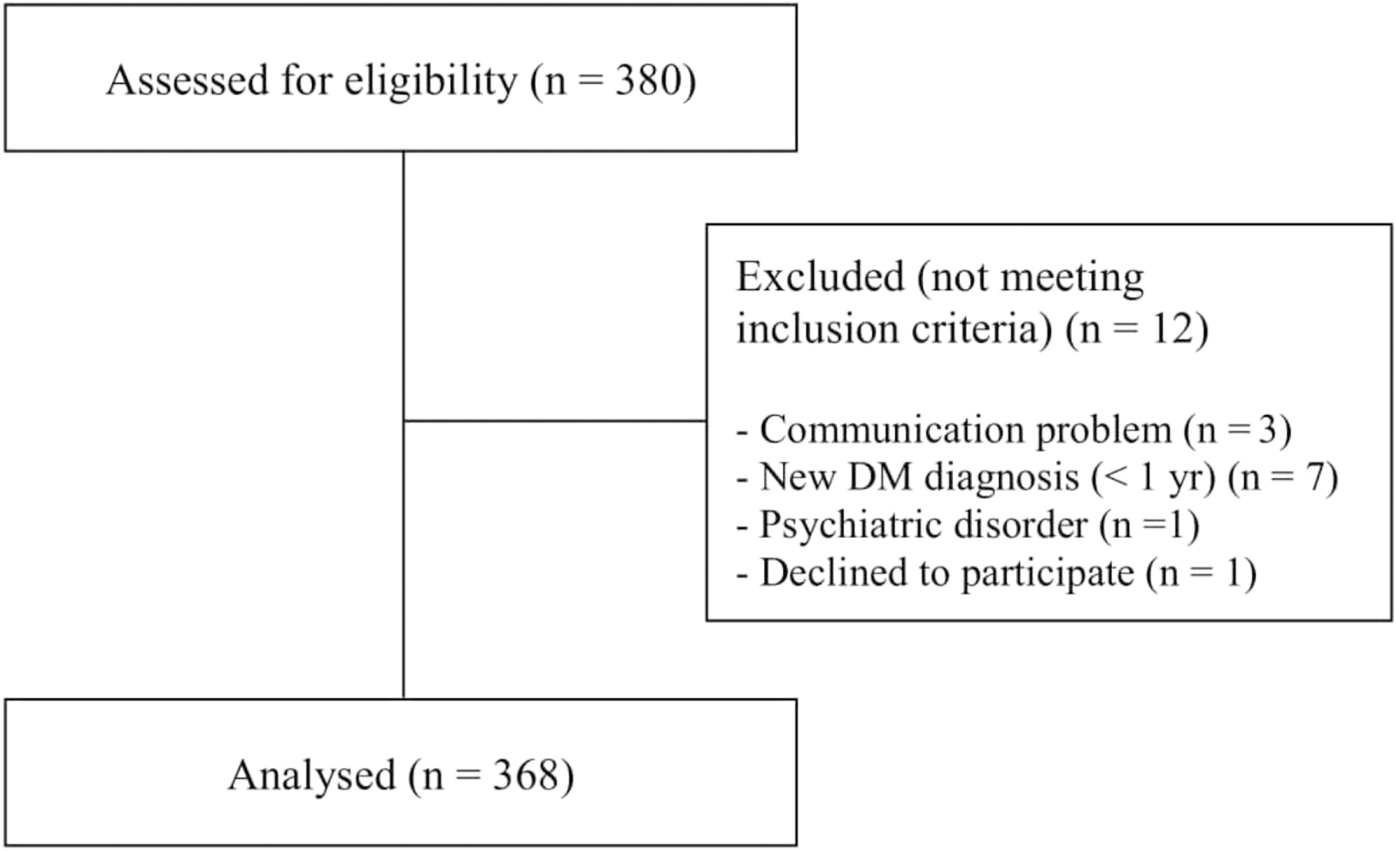
STROBE flowchart for study sample. The figure shows how individuals were included in the study.

Power analysis was used to calculate the sample of the study. Following the power analysis, with 0.99 confidence (α = 0.01), a power of 0.80 (β = 0.20), and medium effect size (0.25), it was determined that 368 participants were needed as a minimum sample size. A total of 380 diabetic patients were seen in the clinics. A total of 368 participants who met the inclusion criteria were included in the study (Fig. 1).

### Data collection and instruments

Data were obtained from the participants via face-to-face interviews and with the use of patient information forms, namely, the foot care behaviour questionnaire (FCBQ), diabetes attitude scale (DAS), illness perception questionnaire (IPQ), health belief model scale (HBMS) and multidimensional scale of perceived social support (MSPSS).

The 22-question patient information form consisted of two parts. The first part consisted of sociodemographic characteristics, such as age, gender and educational status. The second part collected information about the type of diabetes and questions about diabetes, such as training regarding diabetic foot care. Although demographic data were considered self-reported, clinical characteristics (neuropathy and other complications) were obtained from the individual’s medical records.

The FCBQ is a 16-item scale that had been developed to evaluate the diabetic foot care behaviours and was adapted in Turkey by Biçer & Enç (2014). The Turkish version consists of 15 items that are rated as follows: 1 = Never, 2 = Occasionally, 3 = Sometimes, 4 = Frequently and 5 = Always. The maximum score in the Turkish scale is 75 (Biçer & Enç, 2014). The Cronbach’s α coefficient of the Turkish form of the scale was 0.83, and the current study sample was 0.66.

The DAS was developed by the National Diabetes Commission in the United States, and its adaptation in Turkey was implemented by Özcan (1999). The DAS consists of seven subscales, including the need for special education, attitudes towards patient compliance, the seriousness of type 2 diabetes, blood glucose control and complications, the effects of diabetes on patients’ lives, attitudes towards patient autonomy and attitudes towards team care. Items are rated from 1 (strictly disagree) to 5 (strongly agree). In the DAS, a score >3 indicates a positive attitude, and a score ≤3 indicates a negative attitude. Increases or decreases in the scores reinforce shifts in attitude in the respective direction. Meanwhile, in the study of Özcan (1999), the α coefficients were 0.61–0.93, and in the current study, it was 0.73.

The IPQ was adapted in Turkey by Kocaman et al. (2007) and includes disease type (14 common symptoms), opinions about the disease (subscales of time (acute/chronic), outcomes, personal control, treatment control, understanding of illness, duration (cyclical) and emotional representation) and the causes and dimensions of the disease (psychological attributions, risk factors, immune, accidents or chance subscales). In the study, the 38 items of the IPQ and the five-point Likert scale (1-I certainly disagree, 5-I certainly agree) and the performed ‘opinions about disease dimension’ were used. The α-value of the IPQ in the present study was 0.71.

The Turkish form of the HBMS that had been created by Kartal & Özsoy (2014) has five sub-dimensions and a total of 33 items. The sub-dimensions include perceived sensitivity, perceived seriousness, perceived benefits, perceived barriers and health-related recommended activities. A score ≥4 indicates a high/positive health belief, whereas a score <4 indicates a low health belief. The Cronbach’s α coefficient of the Turkish form administered to patients with type 2 diabetes was 0.89. Meanwhile, in the current study, it was 0.83 (Kartal & Özsoy, 2014).

The MSPSS evaluates the adequacy of social supports from three different sources and consists of 12 items. Using a seven-point Likert-type scale (1-certainly disagree, seven-certainly agree), the structured MSPSS consists of three groups related to the sources of the support, which specifically include family, friends and significant others. The maximum possible score is 84, and higher scores indicate high levels of perceived social supports. In that study (Baran, Küçükakça & Ayran, 2014), the reliability coefficients of the scale had high consistency, ranging from 0.80 to 0.95. The α-value of the current study sample was 0.93.

### Data analysis

Statistical evaluation was carried out using the Statistical Package for the Social Sciences software for Windows version 19.0. The mean, standard deviation, median and minimum and maximum values were calculated as descriptive statistics for continuous data, whereas the percentage values were calculated for discrete data. A *t*-test was used to compare the foot care scale with the demographic and clinical variables. Diabetic foot care is a self-care requirement for individuals with diabetes (Ausili et al., 2018). Therefore, the variables examined in the study were determined according to previous studies with self-care behaviours (Ausili et al., 2018; Gurmu, Gela & Aga, 2018; Nie et al., 2018). The Pearson correlation coefficient was calculated on the relationship between demographic and clinical variables and DAS, IPQ, HBMS and MSPSS with FCPQ. The variables that were significant at *p* < 0.05 were included in the regression model. A linear regression analysis was performed to determine the predictors of foot care behaviours.

### Ethical considerations

The Clinical Research Ethics Committee of the University approved this study (31.03.2015/2015/51). A verbal voluntary consent was provided by each patient. Ethical issues (including plagiarism, informed consent, misconduct, data fabrication and/or falsification, double publication and/or submission and redundancy) were monitored by the authors and ethics committee.

## Results

### Demographic and clinical characteristics of the participants

The average age of the participants was 52.4 ± 12.3 years. Most participants were women (57.9%), had been diagnosed with type 2 diabetes (85.3%) for 5–12 years (48.4%), and did not undergo foot examinations (87.5%). However, the participants received training regarding diabetic foot care (42.7%). Most participants had past histories of foot injury (61.4%). Of them, 49.5% had fungal infections. Neuropathy was observed in 10.3% of the patients with diabetic complications.

### Multiple comparisons related to foot care behaviours

The mean FCBQ score of the participants was 54.8 ± 5.0 (min–max = 40–67). Items with the highest average were as follows: ‘After I wash my foot, I dry my toes’ (4.6 ± 0.5), ‘Do not use sharp tools (razor, scissors, etc.) when performing foot care’ (4.6 ± 0.5) and ‘I wear socks that fit my feet, are not too tight or too loose’ (4.6 ± 0.5). Meanwhile, ‘I use moisturising cream on my feet’ had the lowest score (1.5 ± 1.1).

Table 1 depicts individual and clinical characteristics that show significant differences in foot care behaviours. A within‐subject comparison between groups was performed. The average of the participants’ foot care behaviour scores, gender (female; 55.6 ± 5.1, *t* = 2.62, *p* = 0.009), regular exercise (56.5 ± 5.6, *t* = 2.93, *p* = 0.004), diagnosis of type 1 DM (56.4 ± 4.8, *t* = 2.55, *p* = 0.011), history of foot wounds (55.2 ± 5.1, *t* = 1.98, *p* = 0.048) and absence of diabetes-related nephropathy (55.2 ± 5.0, *t* = −3.52, *p* < 0.001) was significantly higher than those within-subjects.

**Table 1:** Comparison of foot care behaviours according to individual characteristics.

Variables*	Foot care behaviour questionnaire		
	Mn ± SD	*t*	*p*
Gender			
Women	55.6 ± 5.1	2.62	0.009
Men	54.2 ± 4.9		
Regular exercise			
Yes	56.5 ± 5.6	2.93	0.004
No	54.4 ± 4.8		
Type of diabetes			
Type-1 DM	56.4 ± 4.8	2.55	0.011
Type-2 DM	54.5 ± 5.0		
Any foot wounds?			
Yes	55.2 ± 5.1	1.98	0.048
No	54.1 ± 4.8		
Nephropathy			
Present	52.6 ± 4.5	−3.52	≤0.001
Not present	55.2 ± 5.0		

**Notes:**

Mn, mean; SD, standard deviation.

* *t*: independent-samples *t-*test.

### Predictors of foot care behaviours

The Pearson correlation test was used to determine variables that were significant predictors of foot care behaviours (Table 2). Age, gender, diabetes type, regular exercise, history of foot wounds, nephropathy, DAS-specific education needs, DAS-blood glucose control and complications, DAS-effect of diabetes on patient life, DAS-attitude towards patient autonomy, IPQ-duration, IPQ-personal control, IPQ-treatment control, HBMS-perceived sensitivity, HBMS-perceived seriousness, HBMS-perceived barriers and HBMS-total score variables were identified as independent predictors. The stated independent variables were tested by multiple regression analysis related to the predictions of participants’ foot care behaviours. Table 3 shows the independent variables with model compatibility (*R* = 0.47, *R*^2^ = 0.22, *F* = 5.694, *p* < 0.001).

**Table 2:** Correlation coefficients on foot care behaviours of individuals.

Variables	Foot care behaviours questionnaire	
	*r*	*p*
Age	−0.12	0.014
Gender	−0.13	0.009
Diabetes type	−0.13	0.011
Regularly exercise	−0.16	0.001
History of foot wounds	−0.10	0.048
Nephropathy	0.18	<0.001
DAS^a^	0.13	0.013
DAS^b^	0.15	0.003
DAS^c^	−0.13	0.003
DAS^d^	−0.12	0.015
IPQ^a^	0.25	<0.001
IPQ^b^	−0.11	0.028
IPQ^c^	0.10	0.041
HBMS^a^	−0.19	<0.001
HBMS^b^	−0.10	0.037
HBMS^c^	−0.20	0.001
HBMS^d^	−0.17	0.001

**Notes:**

DAS^a^, diabetic attitude scale, specific education need subscale.

DAS^b^, diabetic attitude scale, blood glucose control and complications subscale.

DAS^c^, diabetic attitude scale, effect of diabetes on patient’s life subscale.

DAS^d^, diabetic attitude scale, attitude towards patient autonomy subscale.

IPQ^a^, illness perception scale, duration subscale.

IPQ^b^, illness perception scale, personal control subscale.

IPQ^c^, illness perception scale, treatment control subscale.

HBMS^a^, health belief model scale, perceived sensitivity subscale.

HBMS^b^, health belief model scale, perceived seriousness subscale.

HBMS^c^, health belief model scale, perceived barriers subscale.

HBMS^d^, health belief model scale, total scale score.

**Table 3:** Multiple regression analysis of predicting foot care behaviours of individuals.

Predictors	*B*	SE	β	*t*	*p*	*R*	*R*^2^	*F*
Constant (a)	74.71	5.86	0.08	12.73	<0.001	0.47	0.22	5.69
Age	0.03	0.03	0.08	1.05	0.293			
Gender	−1.25	0.49	−0.12	−2.53	**0.012**			
Diabetes type	−1.63	1.23	−0.11	−1.32	0.186			
Regularly exercise	−1.47	0.92	−0.11	−1.59	0.112			
History of foot wounds	−1.46	0.52	−0.14	−2.78	**0.006**			
Nephropathy	2.11	0.71	0.14	2.94	**0.003**			
DAS^a^	−0.42	0.80	−0.03	−0.52	0.598			
DAS^b^	0.81	0.61	0.06	1.32	0.186			
DAS^c^	−1.00	0.50	−0.12	−1.97	0.05			
DAS^d^	−0.53	0.55	−0.06	−0.96	0.374			
IPQ^a^	0.18	0.08	0.14	2.21	**0.027**			
IPQ^b^	−0.22	0.10	−0.11	−2.07	**0.038**			
IPQ^c^	−0.01	0.10	−0.01	−0.10	0.921			
HBMS^a^	−0.98	0.61	−0.11	−1.59	0.111			
HBMS^b^	0.35	0.79	0.03	0.45	0.653			
HBMS^c^	−2.35	1.25	−0.16	−1.87	0.062			
HBMS^d^	−0.34	1.99	−0.02	−0.17	0.864			

**Notes:**

*B*, unstandardized beta; SE, standard error for the unstandardized beta; β, standardized beta; *R*, model; *R*^2^, *R*-squared.

DAS^a^, diabetic attitude scale, specific education need subscale.

DAS^b^, diabetic attitude scale, blood glucose control and complications subscale.

DAS^c^, diabetic attitude scale, effect of diabetes on patient’s life subscale.

DAS^d^, diabetic attitude scale, attitude towards patient autonomy subscale.

IPQ^a^, illness perception scale, duration subscale.

IPQ^b^, illness perception scale, personal control subscale.

IPQ^c^, illness perception scale, treatment control subscale.

HBMS^a^, health belief model scale, perceived sensitivity subscale.

HBMS^b^, health belief model scale, perceived seriousness subscale.

HBMS^c^, health belief model scale, perceived barriers subscale.

HBMS^d^, health belief model scale, total scale score.

Bold indicates that *p* is significant at 0.05 level.

## Discussion

The predictors of foot care behaviours in individuals with diabetes should be identified. Furthermore, follow-up behaviours and follow-up of the development of diabetic foot are important (Neta, Da Silva & Da Silva, 2015; Kurniawan & Petpichetchian, 2011). Our results regarding patient foot care behaviours are important. However, only one study has used the foot care behaviour scale, which is considered reliable in Turkey. Our discussion relative to the existing literature in our country was therefore limited.

Diabetic foot complications may result in diabetic foot ulcers and lower limb amputations, and such complications may affect 15.0–25.0% of patients with all types of diabetes at least once in their lifetime (International Diabetes Federation, 2017; Stevens, Bruneau & Moralejo, 2017; Yudovsky, Nouvong & Pilon, 2010). Compared with individuals without diabetes, those with diabetes are 30 times more likely to undergo lower extremity amputations due to decreased sensory perception and circulatory disturbances that occur secondary to neural damage (International Diabetes Federation, 2017). The damage caused by disease-related metabolic and structural changes can only be reduced by the regular use of effective foot care behaviours (Priya, Nithyaa & PremKumar, 2014; Yudovsky, Nouvong & Pilon, 2010). Foot care may help reduce foot problems by 49.0–85.0% (Bell et al., 2005). In this study, the participants’ FCBQ score was above average, when the maximum score is 75. The participants’ responses to the FCBQ items indicate that their awareness of vascular circulation and prevention of wound development was high. The participants in this study focussed more on correct shoe selection. However, in the study of Chin et al. (2014), foot inspection and use of moisturising lotion predicted the development of diabetic foot ulcers.

In the current study, the foot care behaviours of the female participants were more positive than those of male participants. Several previous studies have shown that, in contrast to the current sample, men exhibited better foot care behaviours than women (Ahmad Sharoni et al., 2017; Muhammad-Lutfi, Zaraihah & Anuar-Ramdhan, 2014). A previous study has emphasized that women spend more time performing daily housework and family care behaviours than men and that they are more likely to neglect foot care behaviours (Ahmad Sharoni et al., 2017), which suggests that the effect of gender observed in the present study was caused by differences in cultural characteristics and differences in diabetic information-seeking behaviours. In this study, gender was a weaker predictor than other variables, despite being a significant predictor of foot care behaviours.

Moreover, the FCBQ scores of the patients with a history of foot wounds were higher than those for within-subject, and this is considered another important finding. Although the literature does not fully support this finding, several assumptions can be made. Those who have a history of foot wounds may have gained foot care behaviours to avoid experiencing such negative conditions again. They may also receive a comprehensive diabetes education (nutrition, foot care, treatment compliance, etc.), as they will be followed-up after hospital admission.

In the present study, gender, history of foot wound, nephropathy, IPQ-duration and IPQ-personal control were significant predictors of foot care behaviour. In particular, gender and history of a foot wound can be attributed to the assumptions described in the previous paragraphs. However, the effect of health belief on foot care behaviours is interesting. In Turkey, no relationship was observed between health beliefs and foot care behaviours in previous studies. Health belief was examined directly using the self-care behaviours of the patients. However, other studies have shown that a good level of health beliefs has a positive effect on the self-care behaviours of individuals with diabetes (Albargawi et al., 2016; Vedhara et al., 2014).

Increasing the level of knowledge and the regulation of comprehensive and scheduled training are the most beneficial interventions for preventing or reducing complications and for the development of foot care behaviours and general health in individuals with diabetes. Those with diabetes who have sufficient information about foot care frequently do not apply this knowledge in their daily lives (Li et al., 2014; Barbui & Cocco, 2002). However, when appropriate diabetic foot care and training were provided, the foot amputation rate decreased by 0.5–0.8% (Leese, Stang & Pearson, 2011). Periodic monitoring and patient education support diabetic foot care behaviours when applied in accordance with pre-existing characteristics, knowledge, attitudes and behaviours (Li et al., 2014; Aypak et al., 2012). In relation to this, longitudinal and interventional studies involving disease perception and foot care attitudes are necessary for preventing the development of foot ulcers. Health professionals should be involved in foot care monitoring and education of individuals with diabetes and, in particular, the attitudes of nurses towards disease and illness should be examined.

## Limitations

The present study had several limitations. Firstly, the FCBQ was previously used in only one study for psychometric and psycholinguistic analyses in Turkey, and it has a limitation in terms of comparing findings using the same scale to those found in our country. Secondly, the study was conducted in a single centre, and this may inhibit the generalization of the results to other populations in Turkey. Thus, further studies of individuals with diabetes must be conducted for comparison and in-depth determination of problems. Another limitation of the study is that the answers to the questions about the foot care behaviour scale were self-reported, and the participants could choose answer based on social desirability.

## Conclusion

The foot care behaviours of individuals with diabetes are influenced by gender, history of foot wound, nephropathy, IPQ-duration and IPQ-personal control. Our results show some factors that may affect diabetic foot care behaviours. Although several of these factors are interchangeable, we cannot say that these alone can be effective in modifying foot care behaviour. Therefore, different variables predicting foot care behaviours should be supported by other studies. In addition, we suggest that health personnel should not ignore the factors associated with diabetic foot care behaviours. In this regard, this study may be a guide for health professionals who are interested in patients with diabetes. Further studies on the role of barriers as predictors of foot care behaviours must be conducted.

## Supplemental Information

10.7717/peerj.6416/supp-1 Supplemental Information 1 STROBE checklist: indicated with line numbers.

10.7717/peerj.6416/supp-2 Supplemental Information 2 SPSS data related to the variables defined in the article are included. In raw data (A) the 1st column shows the subject no; (B) there are sociodemographic and clinical features between the 2nd and 25th columns; (C) FCBQ measures foot care behaviors and is a dependent variable of research; (D) DAS, IPQ, HBMS and MSPSS measure attitude, illness perception, belief, perceived social support of the diabetics, and they are other variables of the research.

## Additional Information and Declarations

### Data Availability

The following information was supplied regarding data availability: The raw measurements are available in the Supplementary File.
